# Did Artificial Intelligence Invade Humans? The Study on the Mechanism of Patients’ Willingness to Accept Artificial Intelligence Medical Care: From the Perspective of Intergroup Threat Theory

**DOI:** 10.3389/fpsyg.2022.866124

**Published:** 2022-05-03

**Authors:** Yuwei Zhou, Yichuan Shi, Wei Lu, Fang Wan

**Affiliations:** ^1^Antai College of Economics and Management, Shanghai Jiao Tong University, Shanghai, China; ^2^Fudan University Sports Medicine Institute, Shanghai, China; ^3^Department of Sports Medicine and Arthroscopy Surgery, Huashan Hospital, Fudan University, Shanghai, China

**Keywords:** artificial intelligence medical care, intergroup threat theory, realistic threats, symbolic threats, willingness to accept (WTA), treatment, diagnosis

## Abstract

Artificial intelligence (AI) has become one of the core driving forces for the future development of the medical industry, but patients are skeptical about the use of AI in medical care. Based on the intergroup threat theory (ITT), this study verified that patients would regard AI as an external group, triggering the perceived threat of the external group, which results in avoidance behaviors in the treatment (experiment 1: *n* = 446) and diagnosis (experiment 2: *n* = 330) scenarios. The results show that despite AI can provide expert-level accuracy in medical care, patients are still more likely to rely on human doctors and experience more negative emotions as AI is more involved in medical care (experiment 1). Furthermore, patients pay more attention to threats at the individual level related to themselves, such as realistic threats related to privacy issues and symbolic threats related to the neglect of personal characteristics. In contrast, realistic threats and symbolic threats at the group level had less effect on patients in the medical scenario (experiment 2).

## Introduction

Artificial intelligence (AI) has been widely used in various medical scenarios such as prevention, diagnosis, and treatment, such as diagnosing heart disease (Feshki and Shijani, 2016), providing medical advice (Nov et al., 2020), detecting skin cancer (Takiddin et al., 2021), identifying layout lesions (Yamashita et al., 2021), and reading CT image of suspected COVID-19 cases (Mei et al., 2020), etc.

The application of AI in the medical field has become a trend. Patients are the end-users of AI health care, and their resistance will directly affect its adoption efficiency (Agarwal et al., 2020). The existing studies show that although the algorithm can be more accurate than doctors (Grove et al., 2000; Eastwood et al., 2012), patients still believe that AI health care cannot provide the same quality of medical care as human doctors (Promberger and Baron, 2006) and cannot be held responsible for errors (Eastwood et al., 2012).

Psychological studies have shown that people are motivated to see their group as distinct from others (Tajfel, 1974). Human-centered attitudes generate negative perceptions of other entities, which include animals, technologies, etc. (Kaplan, 2004; Haslam et al., 2008); that is, people often view other groups as threats (Alexander, 1974; Dunbar, 2013). According to the intergroup threat theory (ITT), by default, humans tend to perceive threats from other groups and show hostility toward them (Stephan and Stephan, 1985). Therefore, we have a reason to believe that people tend to regard AI that does not belong to human groups as a threat, thus causing negative emotions and resistance behaviors(H1).

Furthermore, intergroup anxiety refers to negative emotions generated by interactions with external groups, such as fear, anger, disgust, and hatred. These activated negative emotions will lead to negative behaviors that include avoidance, evasion, resistance, and aggressive (Stephan and Stephan, 2000). In medical services, with the increase in AI involvement, patients will have more contact or interaction with AI that is regarded as outgroup by humans, thus leading to more intergroup anxiety and lower willingness to accept (WTA) it (H2).

Although intergroup anxiety is an important factor in explaining outgroup attitudes. But in subsequent studies, the researchers went a step further and divided the causes of humans’ negative attitudes toward outside groups into the perceived realistic and symbolic threats. Realistic threats include physical harm, loss of authority, or appropriation of resources, whereas symbolic threats refer to the potential challenge to morals, beliefs, and norms caused by groups with different value systems (Stephan et al., 2008, 2009). In addition, these threats revised theory divided into group-level and individual-level threats. The former refers to the threat to the group as a whole, and the latter refers to threats to individual members of the group (Stephan et al., 2008, 2009).

Realistic individual threats concern threats of personal safety, material safety, rights, or general welfare to an individual group member (Stephan et al., 2015). As AI becomes more deeply involved in health care, more and more patients’ personal biometric information is being collected. The personal information may face many potential problems, such as disclosure, misappropriation, or abuse, which poses serious privacy threats to patients and leads to lower WTA (H3).

Symbolic individual threats include the destruction of an individual’s self-identity or self-esteem (Stephan et al., 2015). People have an innate desire to know themselves (Trope, 1975; Baumeister, 2010) and have a perception of whether they have certain characteristics, attributes, abilities, or belonging groups (Kettle and Häubl, 2011). Thereby, when patients believe that AI medical care may ignore their characteristics and unique symptoms, they will be reluctant to use the medical services provided by AI (H4).

Realistic group threats refer to the threat to the rights, resources, and overall welfare of a group, which generally includes political power and economic power (Stephan et al., 2015). People generally regard machines as a threat to human work (Granulo et al., 2019). The existing research shows that there are concerns about AI medical care. People worry that technology-driven productivity gains will lead to redundancies in some healthcare jobs (Hazarika, 2020). When people consider that the emergence of AI will pose a threat to their employment and even affect their future economic situation, they will perceive the group reality threat. As AI becomes more and more involved in medical care, people may worry that AI will replace part of the work of healthcare workers, thus resisting the use of AI health care (H5).

Symbolic group threats include threats to the value system, ideology, and belief system within the group (Stephan et al., 2015). It is predicted that the global AI healthcare market size is expected to grow from $6.9 billion in 2021 to $67.4 billion in 2027, with a CAGR of 46.2%. Key factors driving the growth of the AI healthcare market include the increase in AI tools for care, the increase in the development of AI systems for human perception, and the increasing application of AI technology such as genomics, drug discovery, imaging, and diagnostics in response to COVID-19. Therefore, the widespread use of AI technology will have a certain degree of reform on the medical system. This change will lead to patients’ perception of the threat of AI medical applications and trigger resistance behaviors(H6).

**FIGURE 1:**
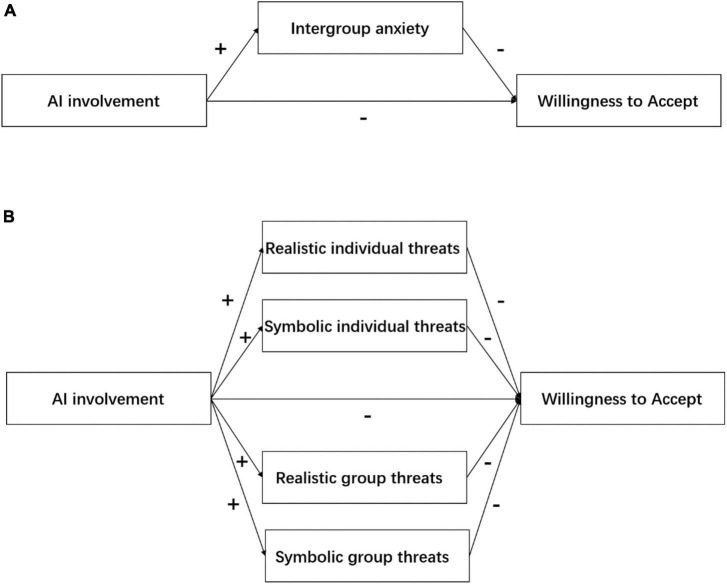
**(A)** The research model of experiment 1. **(B)** The research model of experiment 2.

In summary, we propose the research model shown in Figures 1A,B, which corresponds to the hypotheses shown in Table 1. The purpose of this study is to explore the influence of the degree of AI involvement on patients’ WTA and to reveal whether the reason is caused by patients’ perceived threat to outside groups. In an integrated framework of ITT, the relative influence of different levels of threat on patients’ WTA AI health care is further discussed.

**TABLE 1:** The hypotheses.

Hypotheses
H1: With the increasing AI involvement in medical care, the patients’ WTA decreases
H2: The higher the degree of AI involvement in medical care, the higher the intergroup anxiety patients would feel, which leads to their lower WTA AI medical care
H3: The higher the degree ossf AI involvement in medical care, the higher the realistic individual threats patients would feel, which leads to their lower WTA AI medical care
H4: The higher the degree of AI involvement in medical care, the higher the symbolic individual threats patients would feel, which leads to their lower WTA AI medical care
H5: The higher the degree of AI involvement in medical care, the higher the realistic group threats patients would feel, which leads to their lower WTA AI medical care
H6: The higher the degree of involvement in medical care, the higher the symbolic group threats patients would feel, which leads to their lower WTA AI medical care

## Materials and Methods

### Study Design and Participants

The authors tested all hypotheses in two separate experiments. We recruited participants for two experiments through the online professional research platform Credamo.^1^ All participants were provided with electronic informed consent before participating in the experiment. We promise participants that the experiment survey results will only be used for academic research purposes but not for any commercial purposes, and that the answers involving personal information will be strictly confidential. Participants who agreed continued the experiment, whereas those who refused were allowed to drop out. The experiment collected demographic data of participants, which include age and gender, and personal information, which includes answering duration, IP address, longitude, latitude, province, and city, to ensure the authenticity and validity of the results. Participants in the two experiments were randomly assigned to the between-subjects design to make the groups comparable.

With the most commercially successful precision surgical robots (such as Da Vinci surgical robot) as the background, the experiment 1 explored the influence of AI involvement on patients’ WTA treatment (H1) in high-involvement condition (AI-autonomous treatment), medium-involvement condition (AI-assisted treatment), and low-involvement condition (a human doctor) and examined that the potential cause of patients’ resistance to AI health care is intergroup anxiety (H2).

In experiment 1,446 subjects (median age = 30.4, 65.2% women) were recruited to complete the experiment in exchange for monetary compensation. Participants were randomly assigned into three manipulation conditions and read different descriptions of surgical treatment options relatively (Longoni et al., 2019). Participants in the high-involvement condition read that “surgical scheme will be evaluated by an intelligent program. Using algorithms for comparison. Using minimally invasive stereotactic high-precision surgical robots.” Participants in the mid-involvement condition read that “surgical scheme will be evaluated by surgical specialists with the help of intelligent medical evaluation. Based on clinical experience. Operating highly accurate surgical robot.” Participants in the low-involvement condition read that “the surgical scheme are evaluated by surgical specialists. Based on clinical experience. Cooperating with professional medical team to perform surgery.” All participants were told that surgical robots were as accurate as surgical specialists.

To avoid the particularity of surgical treatment scenarios, in experiment 2, we verified the influence of AI involvement on patients’ WTA in the diagnostic scenario (H1). The mediating effects of realistic individual threats (H3), symbolic individual threats (H4), realistic group threats (H5), and symbolic group threats (H6) were further discussed. The research background of experiment 2 comes from the current genetic testing project based on AI deep learning algorithm which can carry out diagnostic screening that includes genetic diseases, cancer risk, genetic defects, etc. (Longoni et al., 2019).

A total of 330 subjects (median age = 29.2, 64% women) participated in experiment 2 and received monetary compensation. Participants were randomly assigned to read descriptions of two diagnostic screening scenarios with varying degrees of AI involvement (AI-autonomous diagnosis vs. AI-assisted diagnosis). Participants in the AI-autonomous diagnostic condition were highlighted with “analysis based on genetic big data analysis and deep learning algorithms, and assessment reports and detailed health management recommendations generated entirely by advanced AI analysis techniques.” Participants in AI-assisted diagnostic conditions were emphasized that “a professional doctor will evaluate your genetic report and make recommendations based on intelligent test results.”

### Measures

#### Intergroup Anxiety

The measurement of intergroup anxiety was a modified version of the intergroup anxiety scale developed by Stephan and Stephan (1985). In the previous studies, researchers have used this measurement to examine how people feel when interacting with members of other races (Stephan et al., 2002; Tausch et al., 2007). In this study, participants read the following sentence: “I have the following emotions when I think about conducting a surgery by a highly accurate surgical robot/by a surgical specialist with the help of intelligent medical devices/by surgical specialist:” Participants rated how nervous, worried, and afraid they felt using 5-point Likert-type scales (1 = not at all, 5 = very). These items produced a reliable intergroup anxiety index (Cronbach’s α = 0.90).

#### Realistic Individual Threats

As mentioned above, realistic individual threats include threats to an individual’s rights and welfare. Therefore, we used privacy concerns to measure realistic individual threats. The measurement was used in the study of the impact of electronic medical records on patients’ willingness to share personal health data (Cherif et al., 2021). Scale items include “using this medical method would collect too much personal information about me,” “using this medical method would cause my personal data to be disclosed,” “sharing my personal information with other health care providers without my authorization,” and “using my personal data for other purposes without my authorization” (Kim et al., 2008; Ponte et al., 2015). Responses were made on 5-point Likert-type scales ranging from (1) strongly disagree to (5) strongly agree (Cronbach’s α = 0.92).

#### Symbolic Individual Threats

The autonomy of machines has been shown to pose a threat to individual identity and uniqueness (Złotowski et al., 2017). We adopt the uniqueness neglect (Longoni et al., 2019) as a measure of symbolic individual threats. Uniqueness neglect was originally adapted from the personal sense of uniqueness scale (Şimşek and Yalınçetin, 2010). Participants indicated to what extent they agreed with the following statements “the uniqueness of my health condition cannot be recognized,” “my personal special condition will not be considered,” and “no treatment plan can be made based on my special condition” (Cronbach’s α = 0.83). The response format consisted of a 5-point Likert-type scale ranging from strongly disagree to strongly agree.

#### Realistic Group Threats

The measurement method of realistic group threats is adapted from the definition of realistic group threats (Stephan et al., 2002), and the description focuses on the threat of external groups to employment resources and economic resources. This measure has been used by many researchers to examine the realistic threats posed by immigrants and ethnic minorities (Stephan et al., 2002; Tausch et al., 2007). A number of three items were used for the measurement in this study, which include “AI medical care replaced the original job opportunities of doctors,” “AI medical care will lead to a higher unemployment of health care workers,” and “AI medical care will make it more difficult for medical graduates to find jobs” (Cronbach’sα = 0.82). Participants were also rated on a 5-point Likert-type scale, with a higher score indicating a greater perceived threat.

#### Symbolic Group Threats

The measurement of symbolic group threats also refers to the definition of symbolic group threats (Stephan et al., 2002), which focuses on value system and belief. We used a single item “AI medical care will threaten the health care system of our country” for participants to evaluate (1 = strongly disagree, 5 = strongly agree).

#### Willingness to Accept

The measurement of WTA used the methods in the study of understanding, explaining, and utilizing medical AI (Cherif et al., 2021). In experiment 1, participants were asked “How likely would you choose to perform a surgical procedure with a highly accurate surgical robot/surgical specialist using intelligent medical equipment/surgical specialist?” and rated the question on a 5-point Likert-type scale (1 = not at all likely, 5 = very likely). In experiment 2, participants were asked “how likely they are to choose to have health advice provided entirely by AI analytics/human experts with the help of intelligent testing results,” with a score of 1–5 (1 = not at all likely, 5 = very likely).

### Data Analysis

#### Main Effect

In both experiments 1 and 2, one-way ANOVA was used to verify the impact of the degree of AI involvement in medical care on patients’ WTA. The independent variable, the degree of AI involvement, is a categorical variable, which was transformed to a dummy variable at the first.

#### Mediation Effect

To verify the mediating role of intergroup anxiety in the relationship between the degree of AI involvement and patient WTA(H2). We use SPSS (PROCESS Procedure for SPSS version 3.3 is written by Hayes, 2013) to test the mediating effect of multiple categories of independent variables based on the bootstrap (Hayes, 2013).

To verify realistic individual threats (H3), symbolic individual threats (H4), realistic group threats (H5), and symbolic group threats (H6) which mediate the relationship between degree of AI involvement and patient’s WTA, we use SPSS PROCESS Model 4 to conduct a multiple parallel mediation effect test by bootstrap method (Preacher and Hayes, 2004).

## Results

### The Impact of Artificial Intelligence Involvement on Patients’ Willingness to Accept in the Treatment Scenario

Under the same surgical precision, patients have the highest WTA surgical experts (M_low_ = 4.47, *SD* = 0.66), followed by AI as an auxiliary tool (M_mid_ = 3.85, *SD* = 0.89). Patients had the lowest WTA fully autonomous AI health care [M_high_ = 3.40, *SD* = 1.08, *F*(2.42) = 53.00, *p* < 0.001, refer to Table 2].

**TABLE 2:** The impact of the AI involvement on patients’ WTA in the treatment scenario (*n* = 446).

	*N*	*M*	*SD*	SE	95% CI		Min	Max
					Lower	Upper		
Low involvement	148	4.47	0.664	0.055	4.36	4.57	2	5
Mid involvement	150	3.85	0.893	0.073	3.71	4.00	2	5
High involvement	148	3.40	1.080	0.089	3.22	3.57	1	5
Total	446	3.91	0.994	0.047	3.81	4.00	1	5

### The Mediating Role of Intergroup Anxiety

The omnibus test of total effect of AI involvement on patients’ WTA: *F* (2,443) = 53.00 (*p* < 0.001) indicates that the two relative total effects are not all 0. The omnibus test of direct effect of AI involvement on patients’ WTA: *F* (2,442) = 40.23 (*p* < 0.001) indicates that the two relative direct effects are not all 0. Therefore, it is necessary to conduct further relative mediation analysis.

The results of the relative mediation analysis were as follows: with the low involvement as the reference level, the bootstrap confidence interval of 95% of mid involvement was [−0.167, −0.095], excluding 0, which indicates significant relative mediating effect (a_1_ = 0.293, b = −0.285, a_1_b = −0.083). That is, patients’ intergroup anxiety about AI-assisted medical care was 0.293 higher than that of human surgical experts (a_1_ = 0.293). Therefore, the WTA AI-assisted medical care also decreased by 0.530 (c_1_′ = −0.530, *p* < 0.001). The relative total effect was significant (c_1_ = −0.613, *p* < 0.001), and the relative mediating effect was 13.6% (−0.083/−0.613).

**FIGURE 2:**
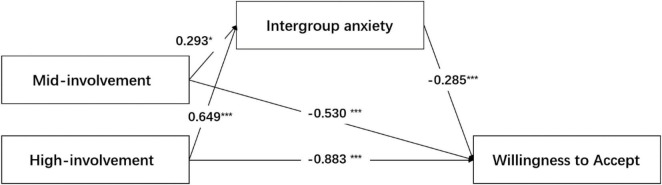
The result of experiment 2. *p < 0.05, ***p < 0.001.

As above, with low involvement as the reference level, the 95% bootstrap confidence interval of high involvement was [−0.285, −0.100], excluding 0, which indicates significant relative mediating effect (a_2_ = 0.649, b = −0.285, a_2_b = −0.185). In other words, patients’ intergroup anxiety about fully autonomous AI medical was 0.649 higher than that of human surgical experts (a_2_ = 0.649), so the WTA fully autonomous AI medical was also reduced by 0.88 (c_2_′ = −0.883, *p* < 0.001). The relative total effect was significant (c_2_ = −1.068, *p* < 0.001), and the relative mediating effect was 17.3% (−0.185/−1.068). The results are shown in Figure 2 and Table 3.

**TABLE 3:** The mediating role of intergroup anxiety (*n* = 330).

Model pathways	Coefficient	Standardized estimate	*p*	95% CI		Ratio of effect
				Lower	Upper	
**Intergroup anxiety on**						
Mid involvement (a1)	0.293	0.136	0.032	0.026	0.559	
High involvement (a2)	0.649	0.136	<0.001	0.381	0.916	
**Willingness to Accept on**						
Intergroup anxiety (b)	–0.285	0.034	<0.001	–0.351	–0.219	
**Relative total effects**						
Mid involvement (c1)	–0.613	0.104	< 0.001	–0.817	–0.409	
High involvement (c2)	–1.068	0.104	< 0.001	–1.373	–0.863	
**Relative direct effects**						
Mid involvement (c1’)	–0.530	0.097	< 0.001	–0.720	–0.339	
High involvement (c2’)	–0.883	0.099	< 0.001	–0.001	–0.688	
**Relative indirect effects**						
Mid involvement (a1*b)	–0.083	0.040		–0.167	–0.010	13.6%
High involvement (a2*b)	–0.185	0.047		–0.286	–0.100	17.3%

### The Impact of Artificial Intelligence Involvement on Patients’ Willingness to Accept in the Diagnostic Scenario

The willingness of patients to accept evaluation reports issued by human experts (M_low_ = 4.05, *SD* = 0.66) is higher than that of big data analysis and deep learning algorithm [M_high_ = 3.63, *SD* = 0.98, *F*(1, 15) = 21.07, *p* < 0.001, refer to Table 4].

**TABLE 4:** The impact of the AI involvement on patients’ WTA in the diagnostic scenario (*n* = 330).

	*N*	*M*	*SD*	*SE*	95% CI		Min	Max
					Lower	Upper		
Low involvement	164	4.05	0.658	0.051	3.95	4.16	2	5
High involvement	166	3.63	0.980	0.076	3.48	3.78	1	5
Total	330	3.84	0.861	0.047	3.75	3.94	1	5

### The Mediating Role of Realistic Individual Threats, Symbolic Individual Threats, Realistic Group Threats, Symbolic Group Threats

**FIGURE 3:**
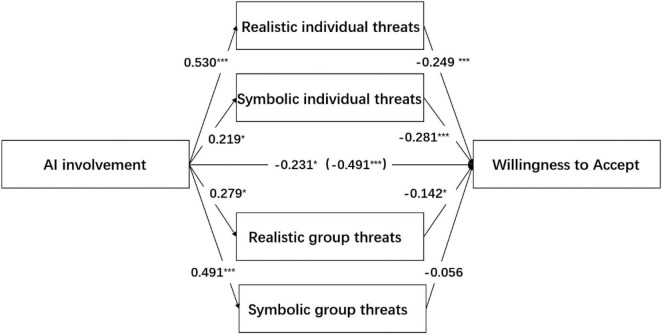
The result of experiment 2. *p < 0.05, ***p < 0.001.

The results of the mediation test showed that the 95% confidence interval (LLCI = −0.220, ULCI = −0.062) of the indirect effect of privacy concern representing realistic individual threats did not contain 0, which indicates the existence of the mediation effect. The 95% confidence interval of the direct effect (LLCI = −0.358, ULCI = −0.039) did not contain 0, which indicates that privacy concern was an incomplete mediator and the mediating effect was −0.132. Similarly, uniqueness neglect that represents symbolic individual threats was as an incomplete mediator (LLCI = −0.137, ULCI = −0.003), with a mediating effect of −0.061. Realistic group threats were incomplete mediation (LLCI = −0.091, ULCI = −0.004), and the mediation effect was −0.040. However, the confidence interval of the indirect effect of symbolic group threats (LLCI = −0.094, ULCI = 0.034) contained 0, which indicates that the mediating effect did not exist. The result is shown in Table 5 and Figure 3.

**TABLE 5:** The mediating role of realistic individual threats, symbolic individual threats, realistic group threats, and symbolic group threats (*n* = 446).

Model pathways	Standardized effect	SE	95% CI		Ratio of effect
			Lower	Upper	
Indirect effects	–0.260	0.065	–0.390	–0.137	53.0%
AI involvement → Realistic individual threats → Willingness to Accept	–0.132	0.040	–0.220	–0.062	26.9%
AI involvement → Symbolic individual threats → Willingness to Accept	–0.061	0.034	–0.137	–0.003	12.5%
AI involvement → Realistic group threats → Willingness to Accept	–0.040	0.022	–0.091	–0.004	8.1%
AI involvement → Symbolic group threats → Willingness to Accept	–0.027	0.032	–0.094	0.034	5.6%
Direct effects	–0.231	0.081	–0.358	–0.039	
Total effects	–0.491	0.092	–0.603	–0.241	

## Discussion

The results of this study showed that patients were reluctant to use AI for medical treatment, even though they knew that AI could provide expert-level accuracy in medical care. The willingness to choose a human doctor is higher than that of AI-assisted medical devices and far higher than that of fully autonomous intelligent medical devices. This means that while AI can provide accurate and effective services in health care, patients are still more likely to rely on human doctors.

According to ITT, people tend to anticipate threats from outside groups, which results in prejudice (Stephan et al., 2005; Morrison et al., 2009; Myers et al., 2013). The study result preliminarily confirmed that people will have intergroup threats when facing AI and resist using AI for medical treatment because of intergroup anxiety. We can see that although the application of AI technology has been all over the medical industry, patients still have a bias against AI medical care.

One of the reasons for this bias and resistance is from realistic individual threats; that is, patients worry about whether there is a privacy leakage and other issues that would harm their rights. Another reason is symbolic individual threats, which means patients generally worry about whether they can receive precise medicine tailored to their condition when using AI medical care. There is also another reason for symbolic group threats related to the wellbeing of healthcare workers. However, symbolic group threats failed to pass the mediation effect verification. From the results, the higher the degree of AI intervention, the more likely patients would perceive the threat to the existing medical system. Although patients have realized that this will have a transformative impact on the healthcare system, there is no significant difference in consumers’ WTA AI as it has been widely used in various healthcare scenarios.

The previous studies have confirmed that people can perceive threats from AI technology in human–computer interaction scenarios. When faced with autonomous AI technology, people experience loss of situational control (Stein et al., 2019), undermining uniqueness of human identity (Złotowski et al., 2017; Stein et al., 2019), feeling security risks (Złotowski et al., 2017), and feeling resource competition (Złotowski et al., 2017), etc.

From the perspective of theoretical contribution, this study explores where patients’ concerns about AI health care come from (which includes individual level, group level, realistic level, and symbolic level) an integrated framework based on ITT. In addition, a test of multiple parallel mediating effects was used to observe the overall mediating effect of all perceived threats and observe the effect of a single threat after eliminating other mediators. Meanwhile, the relative degree of the impact of threats from different levels on patients’ willingness to use AI medical services was also compared.

From the perspective of practical contribution, the research results revealed that patients would have greater realistic individual threats when facing AI health care, such as personal privacy disclosure. The second was symbolic individual threats, such as the lack of precision medical services tailored to the individual’s uniqueness. The least affected were perceived realistic group threats, such as threats to the employment of healthcare workers. When emphasizing the intelligence, accuracy, and cost-effectiveness of AI health care, relevant departments and enterprises may have neglected to understand the sources of negative attitudes and irrational fears about AI health care from the perspective of patients. The research conclusions can help government departments, institutions, and enterprises formulate targeted policies, strategies, or product plans, eliminating patients’ doubts, improving the application of AI in the medical industry, and promoting the benign development of AI health care.

This study has the following limitations. First of all, many other perceived threats can lead to negative attitudes or avoidance behaviors of patients toward AI health care. In this study, only a few representative variables were selected according to the ITT theory to measure threats from different levels. Second, only a single item was used to measure symbolic group threats. These problems need to be further explored by developing more comprehensive scales in the future studies. Third, the research was only conducted in China, so whether there are significant differences in research results under different social cultures needs to be further explored.

Despite these limitations, our research reveals the psychological mechanism of patients’ resistance to the use of AI health care, which deepens the understanding of the current AI medical application. The research conclusions can provide guidance for the application and development of AI in the medical field and provide a reference for policy-making of relevant departments and product promotion of relevant enterprises.

## Conclusion

The study suggests that patients experience intergroup anxiety in the face of AI health care and will resist using AI health care because of the perceived threat. Threats from different levels have a different extent of impacts on patients. In healthcare scenarios, patients first pay attention to threats related to themselves at the individual level, such as personal safety and personal rights. Moreover, patients are more affected by realistic threats (such as personal privacy disclosure) than symbolic threats (such as ignoring individual uniqueness). In contrast, group-level threats have less impact on patients.

## Data Availability Statement

The raw data supporting the conclusions of this article will be made available by the authors, without undue reservation.

## Ethics Statement

The studies involving human participants were reviewed and approved by the Ethics Committee of Science and Technology, Shanghai Jiao Tong University. The patients/participants provided their written informed consent to participate in this study.

## Author Contributions

YZ designed the study and did the writing and analysis, while YS collected data and revised the manuscript. WL gave guidance to the research topic and critically revised the manuscript. FW gave guidance to the research framework. All authors contributed to the article and approved the submitted version.

## Conflict of Interest

The authors declare that the research was conducted in the absence of any commercial or financial relationships that could be construed as a potential conflict of interest.

## Publisher’s Note

All claims expressed in this article are solely those of the authors and do not necessarily represent those of their affiliated organizations, or those of the publisher, the editors and the reviewers. Any product that may be evaluated in this article, or claim that may be made by its manufacturer, is not guaranteed or endorsed by the publisher.

## Footnotes

1
https://www.credamo.com/
